# Supplementary material for: Maize Autophagy-Related Protein ZmATG3 Confers Tolerance to Multiple Abiotic Stresses
Source: Plants (Basel). 2024 Jun 13;13(12):1637. doi: 10.3390/plants13121637 (PMC11207562; doi:10.3390/plants13121637)
Supplement: Supplementary file 1 [file plants-13-01637-s001.zip › plants-3014025-supplementary-figures.pdf]

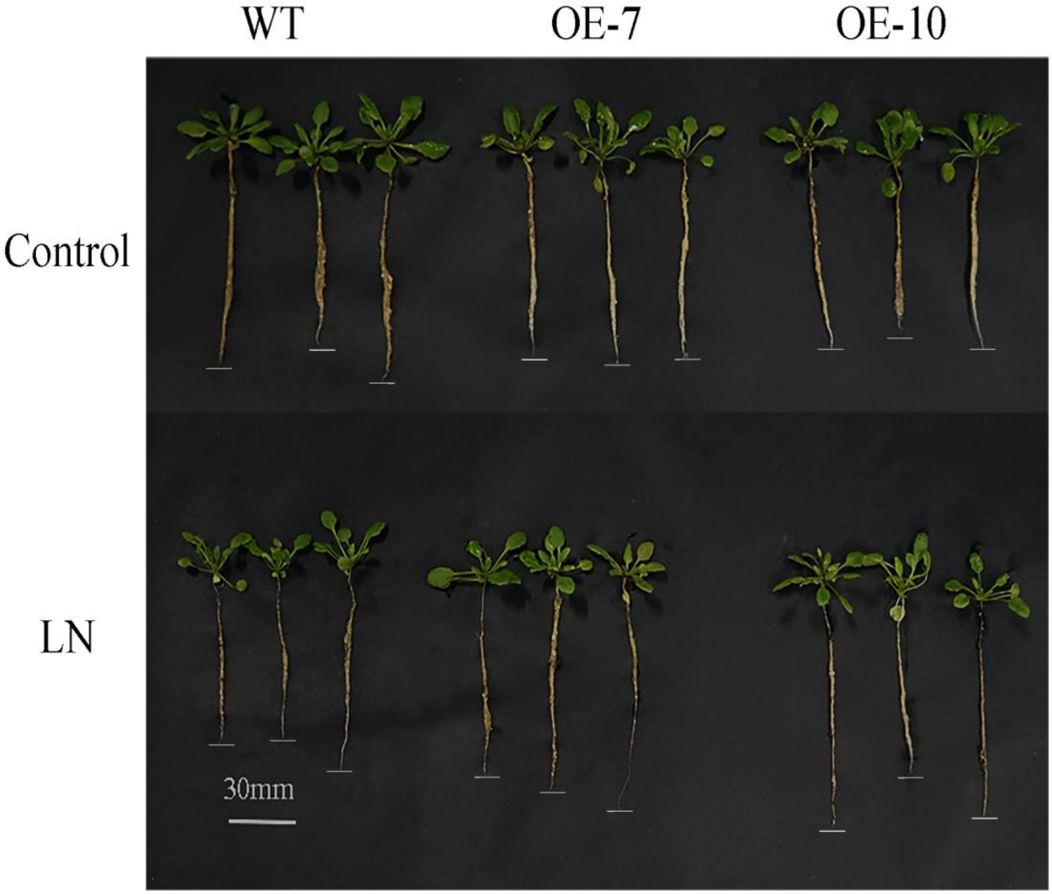

Figure S1. The growth performance between *ZmATG3*-OE (OE) and wild-type (WT) Arabidopsis plants grown under different N levels.

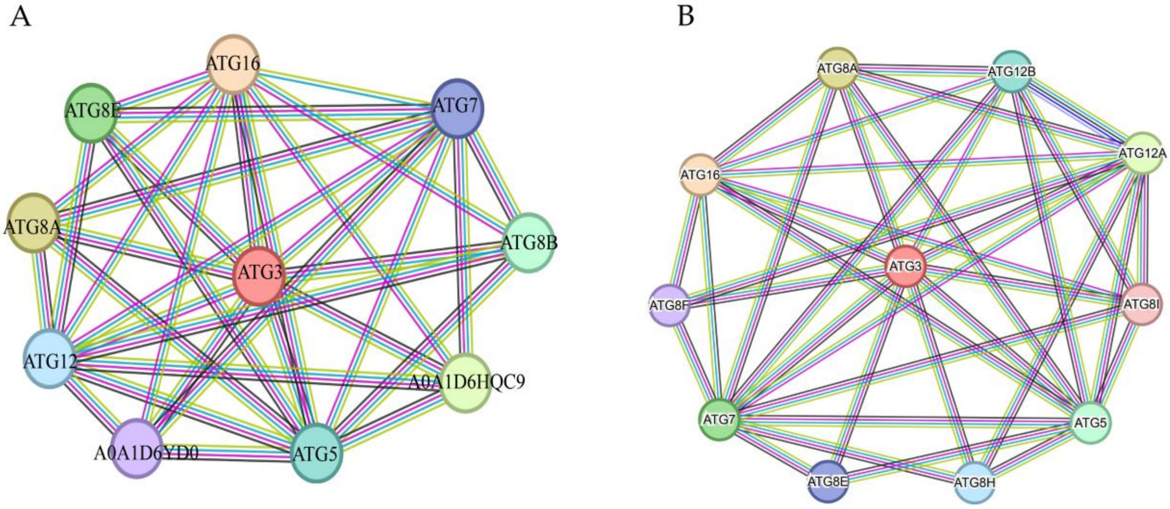

Figure S2. Prediction of proteins interacting with ATG3.
